# Anthropometric predictors of incident type 2 diabetes mellitus in Iranian women

**DOI:** 10.4103/0256-4947.51788

**Published:** 2009

**Authors:** Farzad Hadaegh, Gita Shafiee, Fereidoun Azizi

**Affiliations:** From the Prevention of Metabolic Disorders Research Center, Research Institute for Endocrine Sciences, Shahid Beheshti University of Medical Sciences, Tehran, Iran

## Abstract

**BACKGROUND AND OBJECTIVES:**

Studies have shown a strong association between excess weight and risk of incident diabetes in Iranian women. Therefore, we investigated anthropometric indices in the prediction of diabetes in Iranian women.

**SUBJECTS AND METHODS:**

We examined 2801 females aged ≥220 years (mean [SD] age, 45.2 [12.9] years) in an Iranian urban population who were non-diabetic or had abnormal glucose tolerance at baseline. We estimated the predictive value of central obesity parameters (waist circumference [WC], waist-to-hip ratio [WHR], waist-to-height ratio [WHtR], body mass index [BMI]) in the prediction of diabetes. We classified each parameter in quartiles and compared the lowest with the highest quartile after adjusting for confounding variables, including age, hypertension, triglyceride levels, HDL-cholesterol, family history of diabetes, and abnormal glucose tolerance in a multivariate model. Receiver operator characteristic (ROC) curves were used to determine the predictive power of each variable.

**RESULTS:**

Over a median follow up of 3.5 years (11 months-6.3 years), 114 individuals developed diabetes (4.1%). The risk for developing diabetes was significantly higher for the highest quartile of BMI, WC, WHR and WHtR, respectively, compared to the lowest quartile, and the risk decreased but remained statistically significant when abnormal glucose tolerance was included in the multivariate model. WHtR had the highest area under the ROC curve.

**CONCLUSIONS:**

In Iranian women, BMI, WC, WHR, WHtR were predictive of development of type 2 diabetes, but WHtR was a better predictor than BMI.

Obesity, which increases the risk of coronary heart disease, stroke and type 2 diabetes mellitus (DM), is an important determinant of health.^1^,^2^ The prevalence of obesity and overweight is increasing in developing countries, including Iran.^1^,^2^ DM receives more attention than other related diseases both clinically and in public health.^3^ A prospective epidemiological study showed that increased abdominal fat accumulation is an independent risk factor for cardiovascular disease.^4^ Some studies have suggested that waist circumference (WC) is a better predictor for DM than other indicators of obesity.^5^,^6^ Others have shown that the waist-to-hip ratio (WHR) is the best predictive anthropometric variable for development of type 2 DM.^7^,^8^ In a recent meta-analysis, Vazquez et al showed that body mass index (BMI), WC and WHR had a similar association with incident diabetes.^9^ The ability of obesity indicators to predict diabetes may differ by ethnicity, age and sex.^10^,^11^ Our recent study in Iran showed that incident type 2 diabetes is largely attributable to being overweight, particularly in women.^12^ In the Pima Indian population, BMI and waist-to-height ratio (WHtR) in men, and BMI, WC and WHtR in women were the best predictors of incident diabetes.^13^ Recently, we showed that WHtR was better than BMI in identifying men at risk of diabetes.^14^ This study was designed to determine the best anthropometric predictor of diabetes in a population-based study in urban Iranian women.

## METHODS

This study was conducted within the framework of the Tehran Lipid and Glucose Study (TLGS), a prospective study conducted on a representative sample of residents of district 13 of Tehran (the age distribution and socioeconomic status of the population in district 13 is representative of the overall population of Tehran), with the aim of determining the prevalence of non-communicable disease risk factors and developing a healthy lifestyle to improve these risk factors.^15^ In the TLGS, 15 010 people aged 3 years and older living in district 13 of Tehran were selected by a multistage cluster random-sampling method.^15^ They included 10 368 subjects aged ≥20 years evaluated in the cross-sectional phase 1 of TLGS. Phase 1 was a cross-sectional prevalence study of non-communicable diseases and associated risk factors implemented from March 1999 to December 2001. Phase 2 was a prospective follow-up study which had begun from 2002 to 2005, aiming to determine the trend of non-comunicable disease risk factors and incidence in a representative population. By the end of September 2005, 6246 individuals (59% females and 41% males) had participated in phase 2 of TLGS with a median follow-up duration of 3.5 years (11 months-6.3 years). From this population, 743 with diabetes (271 subjects with current use of a hypoglycemic agent and 472 with newly diagnosed diabetes according to the oral glucose tolerance test results [OGTT]) and 448 with missing data were excluded. Subjects with other forms of glucose intolerance such as impaired GTT or impaired fasting glucose were not excluded. Thus, from 5055 non-diabetic subjects (2085 males and 2970 females) at baseline, 2801 females with full data were included in this study. The main reasons for lack of attendance at follow up examinations despite repeated calls were either immigration (30%) or personal reasons. The Ethical Committee of The Endocrine Research Center of Shahid Beheshti University of Medical Sciences approved the protocol for this study. Informed written consent was obtained from all subjects.

Subjects in each phase were interviewed privately and face-to-face by trained interviewers using pre-tested questionnaires. Initially, information on age, smoking habits, family history of diabetes, and medication use was collected. Subjects who reported a parent or sibling with diabetes were considered to have a positive family history of diabetes and those with a current or past history of smoking were designated as smokers. Weight was recorded to the nearest 100 grams while minimally clothed without shoes using digital scales. Height was measured in a standing position, without shoes, using a tape stadiometer with a minimum measurement of 1 mm, while the shoulders were in a normal state. BMI was calculated as weight in kilograms divided by height in meters squared. WC was recorded to the nearest 0.1 cm at the umbilical level and hip circumference at the maximal level over light clothing, using an unstretched tape meter, without pressure on the body surface. WHR was calculated as WC divided by hip circumference and WHtR as WC (cm) divided by height (cm). To avoid interobserver error, all measurements were taken by the same person. After the patient rested for 15 min, a qualified physician measured blood pressure, taking two measurements (one initial measurement for determining the peak inflation level) in a seated position using a standard mercury sphygmomanometer. There was at least a 30-second interval between these two separate measurements, and thereafter the mean of the two measurements was considered the participant's blood pressure. At baseline and at each phase of the study, a blood sample was taken after a 12-14 hour overnight fast. Blood samples were taken in a sitting position according to the standard protocol and centrifuged within 30-45 min of collection. All blood analyses were done at the TLGS research laboratory on the day of blood collection. For the oral glucose tolerance test (OGTT), 82.5 g of glucose monohydrate solution (equivalent to 75 g anhydrous glucose) was administered orally to all subjects in each phase (excluding those with current use of a hypoglycemic agent) and plasma glucose was measured 2 hours later. The analysis of samples was performed using the Selectra 2 auto-analyzer (Vital Scientific, Spankeren, Netherlands). Fasting plasma glucose (FPG) and 2-hour post-load glucose (2hPG) were measured on the day of blood collection by the enzymatic colorimetric method using glucose oxidize. For lipid measurements, total cholesterol (TC) and triglyceride (TG) kits (Pars Azmoon Inc., Iran) were used. TC and TG were assayed using enzymatic colorimetric tests with cholesterol esterase and cholesterol oxidase, and glycerol phosphate oxidase, respectively. HDL-cholesterol (HDL-C) was measured after precipitation of the apolipoprotein B containing lipoproteins with phosphotungistic acid. All samples were analyzed when internal quality control met the acceptable criteria. Inter-and intra-assay coefficients of variation were 0.5% and 2 for TC and HDL-C and 0.6% and 1.6 for TG, respectively.

### Definition of variables and outcomes

Based on the fasting and 2-hour plasma glucose (2hPG) results, subjects were categorized according to American Diabetes Association (ADA) criteria as having impaired fasting glucose (IFG) (100 mg/dL ≤FPG <126 mg/dL), impaired glucose tolerance (IGT) (140 mg/dL ≤2hPG <200 mg/dL), or diabetes (current use of hypoglycemic agent or FPG ≥126 mg/dL and/or 2hPG ≥200 mg/dL. Abnormal glucose tolerance was defined as having IFG or IGT.^16^ Hypertension was defined as a systolic blood pressure ≥140 mm Hg and/or diastolic blood pressure ≥90 mm Hg, or current use of an antihypertensive medication based on JNC7 (Joint National Committee 7).^17^ BMI was categorized according to WHO recommendations as overweight (BMI=25-<30) and obese subjects (BMI≥30).^18^

### Statistical Analysis

Baseline variables were presented by follow-up diabetes status. Data with normally distributed parameters are presented as means and standard deviations, whereas values for trigylcerides (TG) were log-transformed because of a skewed distribution and expressed as a geometric mean. The mean value and proportions of the baseline variables were compared between subjects who developed diabetes and those who did not using the *t* test and chi-square test, respectively. To identify predictive factors for FPG over the period of follow-up, multiple linear regression analysis was carried out. A logistic regression analysis using a stepwise conditional method was used to calculate the odds ratio (OR) and 95% confidence intervals (CI) for incident diabetes associated with quartiles of anthropometric variables in 2 models: Model 1 was a multivariate model adjusted for age, family history of diabetes, hypertension, HDL-C and TG. Model 2 was a full model, adjusted for the previous variables plus abnormal glucose tolerance at the time of enrollment, considering that the latter is an important risk factor for diabetes. In each model, the subjects were categorized according to their WC, WHR, WHtR, and BMI quartiles. The first quartile was considered as a reference category with DM as outcome variable. Receiver operator characteristic (ROC) curves were used to compare the predictive power of each anthropometric variable after adjustment for age. All the statistical analyses except area under ROC comparisons were performed by SPSS 11.5 software package. The STATA software package version 8 was used to calculate the ROC curve of each anthropometric variable and 95% confidence intervals. *P* values (2-sided) less than.05 were considered statistically significant.

## RESULTS

The mean (SD) age of the women was 45.2 (12.9) years. Incident diabetes was diagnosed in 114 of participants (4.1%, 114/2801) at a median follow up 3.5 years (11 months to 6.3 years). We diagnosed diabetes in 15 subjects by FPG, in 53 subjects by 2hPG, in 19 subjects by both FPG and 2hPG, and in 27 subjects by noting use of hypoglycemic agents. In comparison to the subjects who did not attend the follow-up visit, those who attended had higher baseline values for age (42.6 vs. 40.2 years), BMI (27.6 vs. 26.8 kg/m^2^), WC (88.4 vs. 86.5 cm), WHR (0.84 vs. 0.83), and WHtR (0.56 vs. 0.55) (*P*<.05 for all comparisons). However, the prevalence of hypertension and a family history of diabetes and the mean level of TG and HDL-C were not different between participants and nonlparticipants.

The baseline characteristics of the study subjects according to their follow-up diabetes status are shown in Table 1. Subjects who had developed diabetes at follow up had a significantly higher age, BMI, WC, WHR, WHtR, and a higher level of TG as well as lower HDL-C concentrations than nondiabetics. Diabetic women also had a higher prevalence of hypertension and a positive family history of diabetes, IGT and IFG. Smoking status was not significantly different in those who developed diabetes compared with those who did not develop diabetes. Each anthropometric index explained only about 11% of the variance in FPG after follow-up in a multiple regression analysis (Table 2). When baseline FPG was added to this analysis, this variance increased by about 17% (R^2^=28%) (data not shown). In Table 3 the estimated OR and 95% CI for incident diabetes by quartiles of the anthropometric variables are presented for the two logistic regression models before and after adjustment for abnormal glucose tolerance. In the logistic regression analysis, the ORs (and 95% CIs) in model 1 were 4.8 (2.1-1.10), 6.7 (2.6-6.17), 8.7 (3.0-0.24), and 8.0 (3.1-20.6) for the fourth quartile versus the first quartile for BMI, WC, WHR and WHtR, respectively. Also, the OR of incident diabetes increased across all quartile of anthropometric indices (*P* for trend <.001). After further adjustment for abnormal glucose tolerance (model 2) the OR (95% CI) of the highest quartile of BMI, WC, WHR and WHtR, decreased to 3.1(1.3-7.2), 3.1(1.1-1.8), 4.0, 3.3 respectively, compared to values in model 1, but remained significant. However, the OR for incident diabetes increased across all quartiles of anthropometric indices in the second model (*P* for trend <.05), except for WHR which remained marginally significant (*P* for trend=0.05).

**Table 1:** Baseline characteristics by follow-up diabetes status.

Variable	Diabetic (n=114)	Nondiabetic (n=2687)	*P*
Age (years)	47.5 (11.98)	41.1 (12.7)	<.001
Body mass index (kg/m^2^)	30.3 (4.3)	27.4 (5.1)	<.001
Waist circumference (cm)	95.9 (9.7)	87.2 (12)	<.001
Waist-hip ratio	0.89 (0.06)	0.83 (0.08)	<.001
Waist-height ratio	0.61 (0.06)	0.55 (0.08)	<.001
HdL-C (mg/dL)	40.9 (9.0)	45.4 (11.2)	<.001
Triglycerides (mg/dL)	181 (1.67)	129 (1.70)	<.001
Family history of diabetes (n, %)	49 (43)	722 (26.9)	<.001
Hypertension (n, %)	47 (41.1)	508 (18.9)	<.001
Smoking (n, %)	7 (6.1)	109 (4.0)	.2
Impaired glucose tolerance (n, %)	74 (64.9)	352 (13.1)	<.001
Impaired fasting glucose (n, %)	66 (57.9)	321 (11.9)	<.001

Data are mean (SD) (geometric mean for triglycerides) or number (percent). HDL-C: HDL-cholesterol; family history of diabetes: having a parent or sibling with diabetes; hypertension: blood pressure ≥140/90 mm Hg or usage of antihypertensive agents; smoking: being either current or ex-smoker.

**Table 2:** Multiple linear regression analyses between anthropometric and other independent variables with fasting plasma glucose as dependent variable.

	Body mass index			Waist circumference			Waist-hip ratio			Waist-height ratio		
	B	SE	*P*	B	SE	*P*	B	SE	*P*	B	SE	*P*
Anthropometric variables	0.35	0.05	<.001	0.17	0.02	<.001	15.71	3.50	<.001	23.44	3.53	<.001
Age (years)	0.17	0.02	<.001	0.14	0.02	<.001	0.160	0.02	<.001	0.14	0.23	<.001
Family history of diabetes	1.90	0.52	<.001	1.80	0.52	.001	1.94	0.53	<.001	1.80	0.53	.001
Hypertension	1.06	0.66	0.1	1.08	0.66	0.1	1.39	0.66	.03	1.09	0.66	.1
Triglycerides	0.01	0.003	<.001	0.01	0.003	<.001	0.01	0.003	<.001	0.01	0.003	<.001
HDL-C	−0.07	0.02	.001	−0.07	0.02	.002	−0.08	0.02	.001	−0.07	0.02	.001

	R^2^=0.113			R^2^=0.113			R^2^=0.102			R^2^=0.110		

*Median follow-up 3.5 years (11 months−6.3 years): Anthropometric variables (body mass index, waist circumference, waist-to-hip ratio, waist-to-height ratio) each included separately in four different models.

**Table 3:** Odds ratios (95% confidence intervals) for incident diabetes according to quartiles of anthropometric variables before and after abnormal glucose tolerance adjustment.

Variables	Quartiles	Diabetes (%)	Model 1^a^ OR (95% CI)	*P* for trend	Model 2^b^ OR (95% CI)	*P* for trend
Body mass index (kg/m^2^)	16.2-24.4	1.0	1.0	.001	1.0	.01
	24.5-27.4	3.5	2.6 (1.1-6.1)		1.8 (0.7-4.5)	
	27.5-30.5	3.6	2.2 (0.9-5.3)		1.6 (0.6-4.0)	
	30.6-48	8.2	4.8 (2.1-10.9)		3.1 (1.3-7.2)	

Waist circumference (cm)	58-79.9	0.7	1.0	.001	1.0	.04
	80-86.9	2.7	3.2 (1.2-8.9)		2.2 (0.7-6.3)	
	87-95.9	5.5	5.7 (2.2-14.8)		3.7 (1.4-9.9)	
	96-130	7.3	6.7 (2.6-17.1)		3.1 (1.1-8.3)	

Waist-hip ratio	0.57-0.78	0.6	1.0	.001	1.0	.05
	0.79-0.83	2.8	4.0 (1.3-12.1)		2.6 (0.8-8.1)	
	0.84-0.89	5.6	6.9 (2.4-19.7)		3.6 (1.2-10.7)	
	0.90-1.11	7.5	8.7 (3.0-24.7)		4.0 (1.3-11.8)	

Waist-height ratio	0.36-0.50	0.7	1.0	.001	1.0	.01
	0.51-0.55	2.1	2.4 (0.8-6.7)		1.4 (0.5-4.2)	
	0.56-0.61	5.2	5.1 (1.9-13.4)		2.7 (1.0-7.4)	
	0.62-0.84	8.7	8.0 (3.1-20.6)		3.3 (1.2-8.8)	

a Model 1: multivariate logistic regression model adjusted for age, hypertension, family history of diabetes, HDL-C and TG;

b Model 2: full model adjusted for variables in model 1 plus abnormal glucose tolerance.

**Figure 1:**
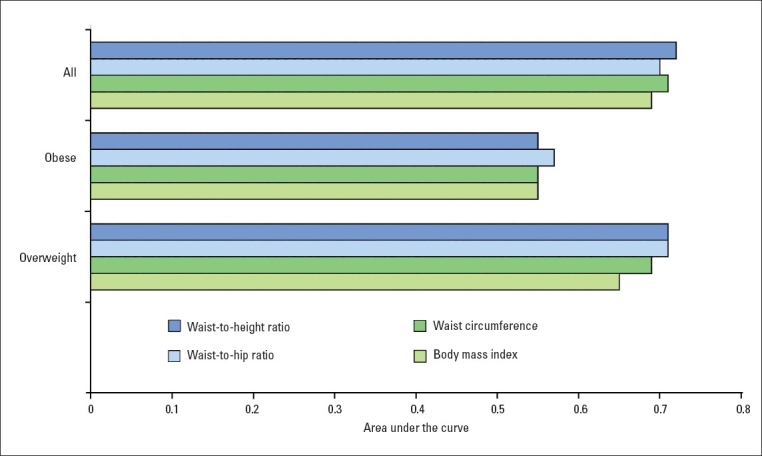
Area under the ROC curve for anthropometric variables in predicting diabetes after age-adjustment. BMI: body mass index, WC: waist circumference, WHR: waist.to–hip ratio, WHtR: waist-to-height ratio, Overweight: BMI=25-29.9 kg/m^2^, Obese: BMI≥30 kg/m^2^. * *P*<.05 compared to BMI.

The baseline obesity indicators in this study were highly correlated with each other. BMI showed high correlation with WC (r=0.83), WHtR (r=0.83) and modest correlation with WHR (r=0.40). WHtR showed high correlation with both WC (r=0.96) and WHR (r=0.79) and finally WHR showed high correlation with WC (r=0.77) (data not shown). Therefore, because of the problem of co-linearity it was not possible to include them in the same regression model and ROC curve analysis was used to compare the predictive power of the different anthropometric variables. Figure 1 showed that only WHtR had a higher area under the ROC curve than BMI after adjustment for age (0.72 vs. 0.69). WC and WHR were considered equal to BMI in their power to predict type 2 diabetes. When the analysis was restricted to overweight and obese subjects, none of the central obesity indicators was confirmed to be superior to BMI.

## DISCUSSION

This prospective study in Iranian women showed that BMI, WC, WHR and WHtR can predict incident diabetes. Our model identified those with a 3- to 4-fold increase in likelihood of developing diabetes during a median follow up of 3.5 years (11 months to 6.3 years); however, the overall predictive discrimination, (as the area under the ROC curves showed), for diabetes was better for WHtR than BMI.

Excess body fat is a main cause of metabolic disturbances such as type 2 DM.^5^ As a simple and non-invasive method, anthropometric measurements have been used to assess general obesity (BMI) and central obesity (WC, WHR, WHtR).^19^ BMI is reported as an indicator for identifying adults at risk of diabetes in many studies.^13^,^20^ However, it has limitations because it does not distinguish overweight due to excess fat mass from lean mass.^21^ In addition, some studies have shown that a high proportion of abdominal fat, particularly visceral fat, is a major risk factor for type 2 DM.^22^ Therefore, other anthropometric parameters are used to assess excess visceral fat. WC and WHR are frequently used to estimate abdominal adipose tissue. Recently, the Obesity in Asia Collaboration study showed that in white females measures of central obesity (WC, WHR) were more strongly associated with diabetes than BMI.^23^ Also, WC was reported by Ford et al as a better predictor than BMI for prediction of metabolic syndrome, diabetes, cardiovascular disease and all-cause mortality.^24^ However, other reports have shown conflicting results.^7^,^25^ Lakka et al in prospective study, suggested WHR is a better index to predict coronary heart disease than WC and BMI.^26^ Our full model (Table 3) showed that the OR for incident diabetes for the highest quartile versus the lowest quartile was greater for WHR followed by WHtR, WC and BMI, although the confidence intervals are wide and overlapping. In addition, despite a lower correlation with BMI and WC (r=0.40 and r=0.77, respectively), the WHR showed the same ability to predict diabetes as both BMI and WC, as discovered by Vazquez et al in a meta analysis.^9^ However, our data confirmed that none of these anthropometric parameters are good measures for predicting future FPG (even when we considered baseline FPG). Considering the limitations of the OR (or relative risks or hazard ratio) as a method of assessing the importance of risk factors and for a more comprehensive picture of the clinical and public health relevance of anthropometric variables, we used ROC curve analyses to compare the predictive validity of these variables.^27^ Among the central obesity variables only WHtR had a significantly larger area under the ROC curve than BMI. In line with our findings, Lin et al showed that in a Taiwanian population WHtR may be a better indicator for predicting cardiovascular risk factors than WC, WHR and BMI, especially for women.^28^ Also, Lorenzo et al showed that area under the ROC curve for WHtR was better than WC for identifying diabetic women.^29^ In a cross-sectional analysis, Schneider et al showed that WHtR may predict prevalent cardiovascular risk better than BMI, WC, and WHR.^30^

Furthermore, our study highlights that in overweight and obese subjects no central obesity variable is superior to BMI. Other studies suggest a stronger effect of body fat distribution on metabolic abnormality risk in normal-weight individuals compared with overweight or obese subjects.^31^,^32^ Our data indicate that the WHtR appears to be a better predictor of DM risk than BMI in the population of women as a whole. The WHtR is simple to assess and is easier to calculate (no squared term is used in the formula) and WC requires only the removal of clothing around the waist. In addition, waist measurement is more sensitive to diet and exercise than BMI because any increase in muscle mass might cause a slight change in BMI, but result in definite changes in WC and thus in WHtR.

This study had some limitations. First, about 40% of the participants in our baseline cohort were excluded from analysis due to loss at followup. This group was healthier in their baseline characteristics; therefore, we may have overestimated the incidence of diabetes in our population. Second, the duration of follow-up was relatively short. Using a longer term follow up would provide stronger evidence although a similarly short follow up was seen in other studies.^33^,^34^ Finally, since chronic diseases are heterogeneous and multifactorial, factors other than anthropometric variables, such as hereditary factors and menopausal state and lifestyle-related factors, should be considered.^35^ This was the first population-based prospective study in Middle Eastern white women, which enhances the validity of our findings. In conclusion, abdominal obesity as measured by WHtR may be better predictors of type 2 diabetes compared to BMI in Iranian women. These simple, inexpensive and noninvasive measures of abdominal obesity is proposed to be incorporated in type 2 DM risk assessment.
